# Intestinal fungi shape the biology of the mammalian gut

**DOI:** 10.1371/journal.ppat.1014132

**Published:** 2026-04-13

**Authors:** Elena Lindemann-Pérez, J. Christian Pérez

**Affiliations:** Department of Microbiology and Molecular Genetics, McGovern Medical School, The University of Texas Health Science Center at Houston, Houston, Texas, United States of America; Duke University Medical Center, UNITED STATES OF AMERICA

Intestinal fungi shape important aspects of mammalian gut biology, including immune development [1,2], bacterial microbiome assembly [3,4], and metabolism [5]. While still neglected in most microbiome studies, gut fungi have been linked to various human diseases including inflammatory bowel disease [2,6,7], pancreatic cancer [8], and liver disease [9,10], among other conditions. It is clear, then, that the fungal community that inhabits our bodies—often referred to as the mycobiome—plays important roles in health and disease. However, basic features of the intestinal mycobiome, such as which fungal taxa can truly be considered gut residents or how they modulate human traits of interest, have remained underexplored. In this Pearls review, we highlight recent developments that provide some answers to these fundamental questions. We also outline opportunities to advance our understanding of the intestinal fungal community and its interplay with the mammalian host.

## Culturomics reaches human gut fungi

Until recently, gut mycobiome studies relied almost exclusively on Internal Transcribed Spacer (ITS) amplicon sequencing data. This approach, while powerful, often fails to provide resolution beyond the genus level. Perhaps more importantly, the systematic collection and culture of gut fungal isolates—an essential resource to advance mycobiome studies—is not part of ITS sequencing projects. A consequence of this is that the experimental research conducted thus far on intestinal fungi has been restricted to few species which can be readily cultivated under standard laboratory conditions, most of them belonging to the *Candida* genus [11]. Two recent reports [12,13] dramatically change the *status quo* by introducing comprehensive and systematic approaches to cultivate gut fungi.

Yan et al. [12] and Zhou et al. [13] introduce optimized *in situ* cultivation systems for fungi with the goal of culturing and studying these eukaryotic microbes derived from human fecal samples. Yan et al. [12] initiated fungal cultivation from fresh fecal specimens of 135 healthy volunteers using multiple fungus-specific media. Zhou et al. [13] designed a diffusion chamber for *in situ* fungal cultivation from fecal samples. The chamber was surrounded by a filter membrane that trapped fungi inside while still allowing uptake of fecal-derived nutrients. This system was applied to fecal samples from ~100 healthy volunteers of geographically diverse areas in China.

The study by Yan et al. [12] was able to cultivate 206 species of gut fungi that span across 4 subphyla, including Mucoromycota, Basidiomycota, Saccharomycotina (yeasts), and Pezizomycotina (filamentous fungi). The authors went on to sequence 744 fungal isolates and profiled enzymes as well as metabolites in several species, providing a remarkable resource. A shortcoming in this large catalogue of isolates and genomes is that it remains unclear whether the cultivated species really inhabit the human digestive tract or are simply bystanders. The latter can originate in foods and beverages that are fermented with a variety of fungi. For example, *Penicillium* is extensively used in the cheese industry whereas *Saccharomyces cerevisiae*, Baker’s yeast, is used in baking, winemaking, and brewing.

The study by Zhou et al. [13] took a step further after cultivating fungal isolates from human feces. To determine which species could adapt to the intestinal environment, they probed growth at body temperature (37 °C) and under low oxygen tension because the large intestine is nearly oxygen free [14]. Using this strategy to filter out bystanders, they convincingly show that at least a subset of the newly identified fungal species can truly reside in the gut environment. The study provides extensive follow-up on filamentous fungi of the genus *Fusarium*. It is shown that *Fusarium* intestinal isolates, especially *F. foetens*, could colonize the murine gut after a single gavage and that they were well tolerated in mice (*Fusarium* are known to produce mycotoxins that can harm animals, but the enteric strains did not produce these toxins). Furthermore, the authors demonstrate that *Fusarium* is detected in human feces across multiple geographical regions of our planet. These findings firmly establish *Fusarium* as *bona fide* members of the human gut microbiota.

By providing biobanked specimens, genome sequences, and extensive phenotypic profiling, these “culturomics” efforts open the door to more mechanistic studies on how these organisms, either alone or as part of microbial communities in the gut, shape the biology of the human host.

## Diet, metabolic health, and gut fungi

Studies in human cohorts have identified associations between the intestinal mycobiome and obesity-associated diseases including type 2 diabetes and nonalcoholic fatty liver disease (reviewed in [15]). Although a cause-and-effect relationship between obesity and fungi remains to be established, a reasonable starting point is to define the contributions of various gut fungi to energy harvest (*i.e.,* whether the presence of fungi alters the sources of energy available to the host) and host metabolism. Taking advantage of a simplified gnotobiotic mouse model, Gutierrez et al. [5] recently began to explore the role of three fungal species (*Rhodotorula mucilaginosa, Malassezia restricta*, and *Candida albicans*) in obesity and metabolic inflammation. In a previous study by the same authors, these 3 fungi had been identified as core species of the infant gut mycobiome that correlated with body mass index scores from 1 to 5 years of age [16].

**Fig 1:**
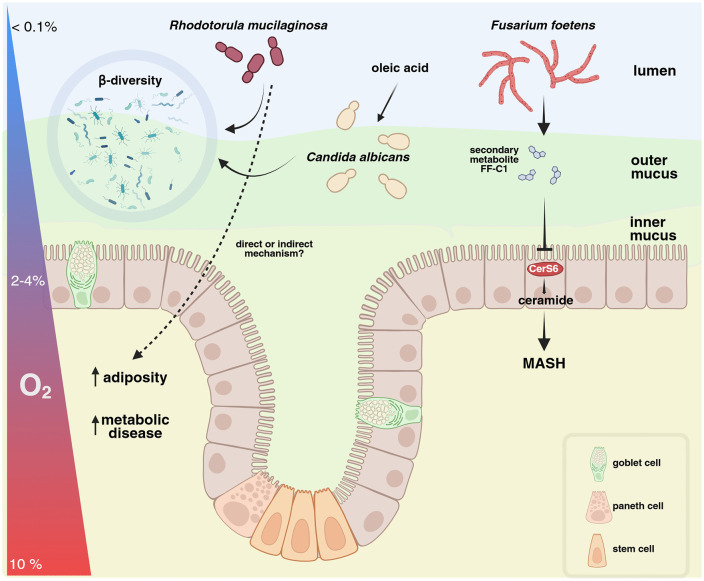
Gut fungi modulate host physiology. Illustrated are three fungal species investigated in the context of intestinal mycobiome-host interactions. Early life colonization of gnotobiotic mice with *Rhodotorula mucilaginosa*, a red-pigmented yeast, alters gut bacterial community composition. In mice fed a high-fat-high-sucrose diet, *R. mucilaginosa* exacerbates metabolic disease, albeit through unknown mechanisms. Dietary oleic acid, one of the most abundant long-chain fatty acids in nature, enhances *Candida albicans* intestinal colonization by promoting modifications to the yeast cell surface that facilitate binding to gut mucin. The filamentous fungus *Fusarium foetens* produces a secondary metabolite that targets an intestinal ceramide synthetase isoform (CerS6), alleviating metabolic disfunction-associated steatohepatitis (MASH) progression in mice. Created in Biorender (https://BioRender.com/o0vu429).

Gutierrez et al. [5] measured multiple parameters of metabolic health in mice under standard or high-fat-high-sucrose diets in the presence of a defined subset of bacteria and individual fungal species. They found that exposure to single fungal species altered the bacterial community composition (*e.g.,* shifts in β-diversity) and had a persistent and substantial impact on fecal metabolome in a species- and diet-specific manner (Fig 1). Colonization with either *C. albicans* or *R. mucilaginosa* had the strongest effects on bacterial abundance, especially on Bacillota including *Limosilactobacillus reuteri and Enterococcus faecalis*. For example, *L. reuteri* relative abundance was reduced upon *C. albicans* or *R. mucilaginosa* colonization in the standard diet. *E. faecalis* abundance increased upon exposure to *R. mucilaginosa* (standard diet) or *M. restricta* (in both diets); however, it was reduced during colonization with *C. albicans* or *R. mucilaginosa* under the high-fat-high-sucrose diets. Besides fungal-induced alterations in the bacterial community structure, fecal metabolome analysis revealed significant metabolome shifts in mice colonized with a defined bacterial community together with *C. albicans* (on both diets) or *R. mucilaginosa* (standard diet). To understand the functional consequences of fungal-induced alterations in the bacterial community, the authors correlated the differential levels of metabolites on either diet with the community metabolic potential of each bacterial species. This analysis suggested that there were direct effects on microbiome metabolic output (e.g., changes in uracil and succinate levels could be ascribed to *C. albicans*-induced shifts in the bacterial community) and effects influenced by the fungi via changes in bacterial species abundance and/or metabolism (e.g., hypoxantine levels in the case of *R. mucilaginosa*).

Colonization with *R. mucilaginosa* and *M. restricta* increased adiposity in mice fed the standard diet; however, only *R. mucilaginosa* colonization led to metabolic disease. Surprisingly, under obesogenic diet conditions, *C. albicans* colonization promoted resistance to diet-induced obesity and led to a lean mouse phenotype. While nutrient absorption in the small intestine remained unaffected, a comprehensive assessment of immune cell population in visceral fat tissue revealed significant changes that varied according to fungal colonization, diet, and sex.

The described study [5] shows unambiguously that gut fungi can alter host metabolic traits in the context of an obesity-inducing diet. A complementary question to be addressed is, what are the effects of dietary components, particularly lipids, on the physiology of the fungal species themselves? A recent report by Shubaita et al. [17] provides clues. This study shows that a diet rich in oleic acid (a monounsaturated long-chain fatty acid) enhances *C. albicans* colonization in the intestine of antibiotic-naive mice (Fig 1). Remarkably, neither changes in the bacterial microbiota nor fungal fatty acid catabolism (via β-oxidation) seemed to mediate the oleic acid effect on colonization. Rather, the authors demonstrate that under anaerobic conditions, dietary oleic acid induces changes to the fungal cell surface which promote stronger interactions between fungal cells and mucin, a key component of the intestinal milieu.

Taken together, these studies underscore the intertwined dependencies between fungal and bacterial species as well as the critical roles that dietary components have in shaping both mycobiome constituents and host metabolism.

## A metabolite derived from a gut commensal fungus targets host lipid homeostasis

Fungi are major and prolific producers of secondary metabolites. It is reasonable to expect, therefore, that human gut mycobiota-derived metabolites impact host intestinal physiology. The field, however, has been slow to show progress in this front, likely because defining physiologically relevant metabolites and targets can be major undertakings. A breakthrough came recently through the identification of a gut commensal fungus-derived metabolite and its target in the mammalian intestine.

Zhou et al. [13] investigated the role of the filamentous fungus *Fusarium foetens* in metabolic disfunction-associated steatohepatitis (MASH). They found that *F. foetens* alleviates MASH progression in mice. The effect was also observed in germ-free mice monocolonized with the fungus, indicating that *F. foetens* directly targets some aspect of host physiology. A screening method to systematically evaluate colonic enzyme activity revealed that *F. foetens* reduced activity of a specific isoform of ceramide synthetase (CerS6), a key enzyme in ceramide metabolism (see [18] for a primer on ceramides and their roles in health and disease). Ceramides are vital components of intestinal epithelial cell membranes and help maintain the physical barrier of the epithelium. Ceramides are composed of sphingosine and fatty acid chains with different chain lengths. The size of the fatty acid chains confers different functions to these lipids. C16:0 ceramide is mainly produced in the gut, but several studies have demonstrated that it can cause liver inflammation and promote the occurrence and development of MASH [19,20]. Intestinal CerS6 is the main enzyme that produces C16:0 ceramide. Through exquisite and extensive follow-up, the authors found that the *F. foetens*-derived secondary metabolite FF-C1 (2,3-dihydro-5-hydroxy-8-methoxy-2,4-dimethyl-naphtho-[1,2-b]-furan-6,9-dione) (a 1,4-naphthoqhinone) can reduce the levels of C16:0 ceramide by inhibiting intestinal CerS6 activity (Fig 1), thus playing a protective role against MASH. While it remains to be seen if the negative correlation between fungus and MASH extends to humans, this constitutes a major advance to both the mycobiota and liver disease fields.

This work demonstrates that human gut mycobiota-derived metabolites target consequential aspects of host intestinal physiology. As an increasing number of human disorders become associated with mycobiota alterations, it may be expected that other fungal secondary metabolites may also shape additional cellular pathways and functions in intestinal cells.

## Culturing gut fungi in anaerobic environments is key to dissect their biology

Investigating the roles that gut fungi have in the host requires not only experiments in mice but also culturing these fungal isolates under defined laboratory conditions. In this context, it is paramount to consider that the large intestine, where these microorganisms are typically at their highest density, is an environment largely devoid of oxygen. A growing body of literature shows, perhaps not surprisingly, that the physiology and behavior of gut commensal fungi, particularly of the genus *Candida*—which have been studied the most—are quite different between aerobic and anaerobic environments. For example, *C. albicans* cultures barely increase their optical density in anaerobic environments [17,21], although cells remain viable for extended periods of time under these conditions [17]. These observations have been made in both defined (yeast nitrogen broth) and complex (Todd Hewitt broth) media formulations; it is plausible, however, that these laboratory media may not accurately represent the milieu that allows fungal species to proliferate under these conditions and that adding certain supplements might enhance anaerobic growth. *C. albicans* transcriptome analyses conducted under anaerobic conditions have found a substantial reduction in transcripts associated with growth and metabolism [22], supporting the idea that actively dividing cells are rare in anaerobic environments. Likewise, body temperature (37 °C) fails to induce the yeast-to-hyphae transition in *C. albicans* under anaerobic conditions [17,23] even though this temperature is a robust hyphal inducer aerobically. Furthermore, the phenotypes ascribed to some gene deletions can differ dramatically depending on the presence of oxygen. For instance, the *C. albicans* transcription factor *HMS1* acts as a positive or negative regulator of filamentation under aerobic or anaerobic conditions, respectively [23].

As the field moves towards defining fungal pathways that drive intestinal colonization and fungal metabolites mediating interactions with the host, culturing gut fungi under anaerobic conditions will be key to pinpoint relevant molecules and mechanisms.

## Investigating fungi that reside in the intestinal mucus layer

The intestinal tract harbors a variety of distinct microenvironments along its longitudinal and cross-sectional axes. Mucus, in particular, creates a physical barrier that effectively partitions the gut lumen from the intestinal surface [24,25]. Accordingly, the luminal and mucosal compartments of the gut constitute utterly divergent microbial niches [26,27]. ITS sequencing-based analysis of the murine mycobiota in these two locations [28] revealed that the intestinal lumen includes genera such as *Cladosporium* and *Aspergillus*, which are likely bystanders, whereas the intestinal mucus-associated taxa are enriched with fungal genera such as *Candida* which has been shown to be a permanent inhabitant of the digestive tract. Consistent with the sequencing data, imaging conducted in gnotobiotic mice also identified a *C. albicans* subpopulation laying in the colonic mucus layer [29].

Because of their proximity to intestinal cells, mucus-embedded fungi are the most likely to elicit, and respond to, host signaling events in the epithelium [28,30]. In fact, *C. albicans* has been found to be the most “immunoreactive” fungal species in the human intestine [1]. Likewise, due to their access to the gut’s lining, the *Candida* cells that traverse the intestinal epithelium and seed disseminated infections are likely to be those residing in the mucus layer. Several transcriptome data sets have been generated from *Candida* in mouse intestinal or cecal contents, revealing important fungal adaptations to colonize the mammalian gut [31,32]. However, it remains unclear whether the same set of genes expressed by the fungus in the intestinal lumen also plays a role when the organism is physically closer to the intestinal epithelium. The notion that gut microbes adjust the repertoire of expressed genes according to the intestinal microenvironment where they reside has been demonstrated for *Bacteroides fragilis. B. fragilis* cells exclusively located in the mucus layer produce a sulfatase (BF3086) and a glycosyl hydrolase (BF3134) required to degrade mucin glycans. These enzymes are essential for colonization because their deletion impairs growth on mucus and reduces the ability of this bacterial species to colonize the host [33]. Bacteroides and other colonic mucosa-associated bacteria, such as *Bifidobacterium* and *Roseburia* also regulate host lipid metabolism by fermenting complex carbohydrates into short-chain fatty acids which can act as signaling molecules to influence epithelial integrity and metabolism. These bacteria directly modulate intestinal barrier function, lipid absorption, and immune responses by interacting with the epithelial layer [34]. Thus, as our understanding of intestinal fungi develops, it is paramount to incorporate tools and methods to dissect the fungal cells occupying the intestinal mucus layer.
